# Identification of the differential expression of genes and upstream microRNAs in small cell lung cancer compared with normal lung based on bioinformatics analysis

**DOI:** 10.1097/MD.0000000000019086

**Published:** 2020-03-13

**Authors:** Xiuwei Li, Chao Ma, Huan Luo, Jian Zhang, Jinan Wang, Hongtao Guo

**Affiliations:** a Department of Radiotherapy, Zhoukou Central Hospital, Zhoukou, China; b Department of Cardiology; c Department of Ophthalmology, Campus Virchow, Charité–Universitätsmedizin Berlin, Berlin, Germany.

**Keywords:** bioinformatics analysis, differential expression, gene, lung cancer, microRNA, small cell lung cancer

## Abstract

Small cell lung cancer (SCLC) is one of the most lethal cancer, mainly attributing to its high tendency to metastasis. Mounting evidence has demonstrated that genes and microRNAs (miRNAs) are related to human cancer onset and progression including invasion and metastasis.

An eligible gene dataset and an eligible miRNA dataset were downloaded from the Gene Expression Omnibus (GEO) database based our screening criteria. Differentially expressed genes (DE-genes) or DE-miRNAs for each dataset obtained by the R software package. The potential target genes of the top 10 DE-miRNAs were predicted by multiple databases. For annotation, visualization and integrated discovery, Metascape 3.0 was introduced to perform enrichment analysis for the DE-genes and the predicted target genes of the selected top 10 DE-miRNAs, including Pathway and Process Enrichment Analysis or protein–protein interaction enrichment analysis. The intersection of predicted target genes and DE-genes was taken as the final DE-genes. Then apply the predicted miRNAs-targets relationship of top 10 DE-miRNAs to the final DE-genes to gain more convinced DE-miRNAs, DE-genes and their one to one relationship.

GSE19945 (miRNA microarray) and GSE40275 (gene microarray) datasets were selected and downloaded. 56 DE-miRNAs and 861 DE-genes were discovered. 297 miRNAs-targets relationships (284 unique genes) were predicted as the target of top 10 upregulating DE-miRNAs. 245 miRNAs-targets relationships (238 unique genes) were identified as the target of top 10 downregulating DE-miRNAs. The key results of enrichment analysis include protein kinase B signaling, transmembrane receptor protein tyrosine kinase signaling pathway, negative regulation of cell differentiation, response to growth factor, cellular response to lipid, muscle structure development, response to growth factor, signaling by Receptor Tyrosine Kinases, epithelial cell migration, cellular response to organic cyclic compound, Cell Cycle (Mitotic), DNA conformation change, cell division, DNA replication, cell cycle phase transition, blood vessel development, inflammatory response, *Staphylococcus aureus* infection, leukocyte migration, and myeloid leukocyte activation. Differential expression of genes-upstream miRNAs (RBMS3-hsa-miR-7–5p, NEDD9-hsa-miR-18a-5p, CRIM1-hsa-miR-18a-5p, TGFBR2-hsa-miR-9–5p, MYO1C-hsa-miR-9–5p, KLF4-hsa-miR-7–5p, EMP2-hsa-miR-1290, TMEM2-hsa-miR-18a-5p, CTGF-hsa-miR-18a-5p, TNFAIP3-hsa-miR-18a-5p, THBS1-hsa-miR-182–5p, KPNA2-hsa-miR-144–3p, GPR137C-hsa-miR-1–3p, GRIK3-hsa-miR-144–3p, and MTHFD2-hsa-miR-30a-3p) were identified in SCLC.

RBMS3, NEDD9, CRIM1, KPNA2, GPR137C, GRIK3, hsa-miR-7–5p, hsa-miR-18a-5p, hsa-miR-144–3p, hsa-miR-1–3p along with the pathways included protein kinase B signaling, muscle structure development, Cell Cycle (Mitotic) and blood vessel development may gain a high chance to play a key role in the prognosis of SCLC, but more studies should be conducted to reveal it more clearly.

## 1 Introduction

Small cell lung cancer (SCLC) is a high grade poorly differentiated neuroendocrine carcinoma of the lung, which represents approximately 15% of bronchogenic carcinomas and up to 25% of lung cancer deaths.^[1,2]^ SCLC is associated with early metastasis and poor patient survival.^[3]^ Regardless of stage, the current prognosis for patients with SCLC is unsatisfactory despite improvements in diagnosis and therapy made during the past 25 years. Without treatment, SCLC has the most aggressive clinical course of any type of pulmonary tumor, with median survival from diagnosis of only 2 to 4 months.^[2]^ Overall survival in SCLC is dismal with a 5-year survival of ∼2% for extensive stage metastatic disease, which comprises 70% of cases at initial diagnosis.^[4]^ SCLC is more responsive to chemotherapy and radiation therapy than other cell types of lung cancer; however, a cure is difficult to achieve because SCLC has a greater tendency to be widely disseminated by the time of diagnosis.^[2]^

In view of the potentially limited impact of the further developments of standard therapeutic regimens on patient survival, the development of targeted therapies based on a better understanding of the molecular basis of the disease is urgently needed.^[5]^ At the genetic level, SCLC appears to be very heterogeneous, although somatic mutations targeting classical oncogenes and tumor suppressors, such as MYC, TP53, and RB1 have been reported, more genes involved in SCLC are waiting for us to explore furtherer.^[5]^ MicroRNAs (miRNAs) are a group of small endogenous single-stranded non-coding RNAs, ∼21 to 25 nucleotides in length.^[6]^ MiRNAs can negatively modulate gene expression via binding to the 3′-untranslated region of messenger RNA (mRNA), thereby leading to direct degradation of mRNA or suppression of protein translation. Through this approach, miRNAs are involved in regulation of many biological processes such as proliferation, apoptosis, cell cycle and differentiation, and DNA repair.^[7]^ Over the past decades, mounting studies have demonstrated that miRNA is frequently abnormally expressed in various types of cancer including SCLC, and the dysregulation of miRNA plays a paramount role in tumorigenesis, invasion and metastasis.^[8,9]^ However, research exploring Differential expression miRNAs in SCLC based on large-scale human tissues are rarely seen.

With the rapid development of gene chip and RNA sequencing technologies, Gene Expression Omnibus (GEO) gradually plays an important role in the bioinformatic analysis.^[10]^ It can provide us with novel clues for discovering reliable genes and miRNAs. In the present study, we explore the differential expression of genes and miRNAs in SCLC and normal lung tissue and the potential molecular mechanisms related to them based on GEO database and comprehensive bioinformatic analysis.

## 2 Materials and methods

### 2.1 MiRNA and gene microarrays

In the discovery step, we log in to the National Center for Biotechnology Information (NCBI) GEO database (https://www.ncbi.nlm.nih.gov/geo) to look for the microarrays we need. We only considered datasets that compared the miRNA and gene expression in SCLC tissue with normal lung tissue. Besides, the containing of samples in such datasets should be over forty. The titles and abstracts of these datasets were screened, and the full information of the datasets of interest was further evaluated. We will choose one most suitable miRNA and gene microarrays respectively.

### 2.2 Screening for DE-miRNAs and DE-genes

Data were normalized using the normalizeBetweenArray function from R package “LIMMA” from the bioconductor project. The miRNA and gene differential expression analysis were conducted using the limma software package in the Bioconductor package (http://www.bioconductor.org/). The related codes were put into R, and the DE-miRNAs and DE-genes in SCLC tumor samples compared to normal lung samples were analyzed through the limma package. FDR (False Discovery Rate) adjusted *P*-value < .001 and |fold change (FC)| > 2 were set as the thresholds for identifying DE-miRNAs and DE-genes. The upregulated or downregulated DE-miRNAs and DE-genes were sorted according to the size of their |fold change (FC)|.

### 2.3 Prediction of target genes for DE-miRNAs

The potential target genes of the top 10 most upregulated and downregulated DE-miRNAs were obtained from the intersection of prediction of miRTarBase Release 7.0 (http://mirtarbase.mbc.nctu.edu.tw), TargetScan Release 7.2 (http://www.targetscan.org) and miRDB Version 5.0 (http://mirdb.org). miRTarBase Release 7.0 is an experimentally validated microRNA-target interactions database, which is updated on September 15, 2017. TargetScan Release 7.2 updated on March 2018, and it is a web server that predicts biological targets of microRNAs by searching for the presence of sites that match the seed region of each miRNA. miRDB Version 5.0 released on August 2014, it is an online database for miRNA target prediction and functional annotations.

### 2.4 Enrichment analysis

The monthly updated database for annotation, visualization and integrated discovery (Metascape 3.0, http://metascape.org) was introduced to perform enrichment analysis for the DE-genes and the predicted target genes of the selected 20 DE-miRNAs, including Pathway and Process Enrichment Analysis or Protein-protein Interaction Enrichment Analysis. All genes in the genome have been used as the enrichment background. Terms with a *P*-value < .01, a minimum count of 3, and an enrichment factor > 1.5 (the enrichment factor is the ratio between the observed counts and the counts expected by chance) are collected and grouped into clusters based on their membership similarities. More specifically, *P*-values are calculated based on the accumulative hypergeometric distribution,^[11]^ and *q*-values are calculated using the Banjamini–Hochberg procedure to account for multiple testings.^[12]^ Kappa scores^[13]^ are used as the similarity metric when performing hierarchical clustering on the enriched terms, and sub-trees with a similarity of >0.3 are considered a cluster. The most statistically significant term within a cluster is chosen to represent the cluster. For each given gene list, protein–protein interaction enrichment analysis has been carried out with the following databases: BioGrid6, InWeb_IM7, OmniPath8. The resultant network contains the subset of proteins that form physical interactions with at least one other member in the list. If the network contains between 3 and 500 proteins, the Molecular Complex Detection (MCODE) algorithm9 has been applied to identify densely connected network components. Pathway and process enrichment analysis has been applied to each MCODE component independently.

### 2.5 Conjoint analysis of DE-miRNAs and DE-genes

In this step, due to the special regulation mechanism of miRNAs, we intersect the upregulated DE-genes and the predicted target genes of top 10 most downregulated DE-miRNAs, apply the prediction miRNAs-targets relationship of top 10 most downregulated DE-miRNAs to the intersection. Then, intersect the downregulated DE-genes and the predicted target genes of top 10 most upregulated DE-miRNAs, apply the prediction miRNAs-targets relationship of top 10 most upregulated DE-miRNAs to the intersection. In order to gain more convincing DE-miRNAs, DE-genes, and their one to one relationship.

### 2.6 Statistical analysis

The results were shown as mean ± SD. Differences between two groups were estimated using unpaired Student's *t* test. A two-tailed value of *P* < .05 or FDR adjusted *P*-value < .05 was considered as statistically significant.

## 3 Results

### 3.1 Microarrays

We found eight datasets regarding normal lung tissue compared with SCLC tissue. But six of them have more smaller simply size, so finally, only GSE19945 and GSE40275 datasets were selected to further study. The dataset GSE19945 was based on the platform of GPL9948 (Agilent Human 0.6K miRNA Microarray G4471A), contained 8 human normal lung samples and 35 human SCLC samples. GSE40275 is a GPL15974 platform (Human Exon 1.0 ST Array)-based dataset, contained 43 human normal lung samples and 15 human SCLC samples (Table 1).

**Table 1:**
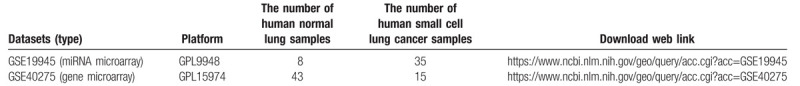
Basic information of the selected datasets.

### 3.2 Identification of DE-miRNAs and DE-genes

**Figure 1:**
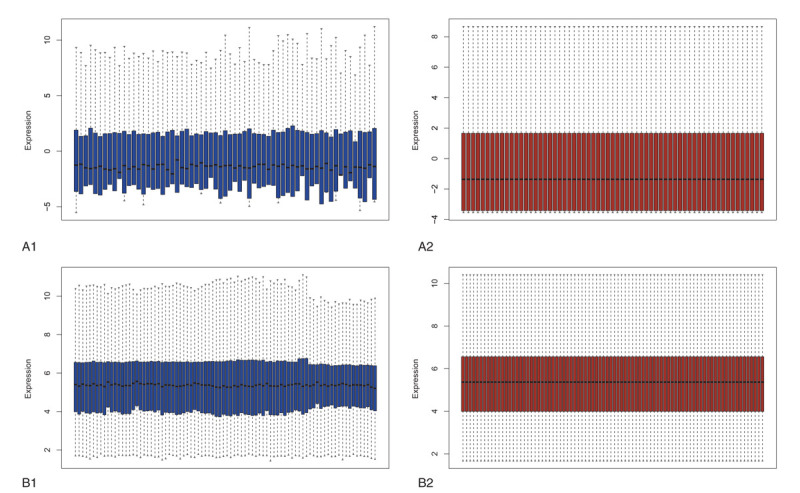
Normalization of datasets GSE19945 (A) and GSE40275 (B). (A1 and B1) Data before normalization; (A2 and B2) data after normalization.

**Figure 2:**
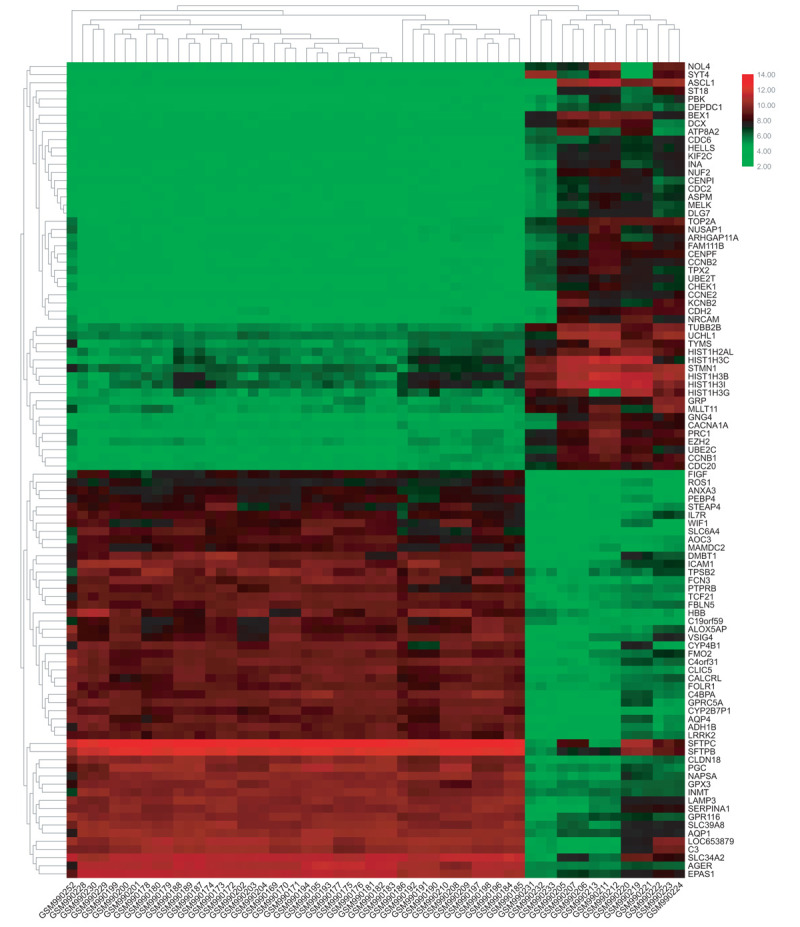
The heatmap of differentially expressed genes.

**Figure 3:**
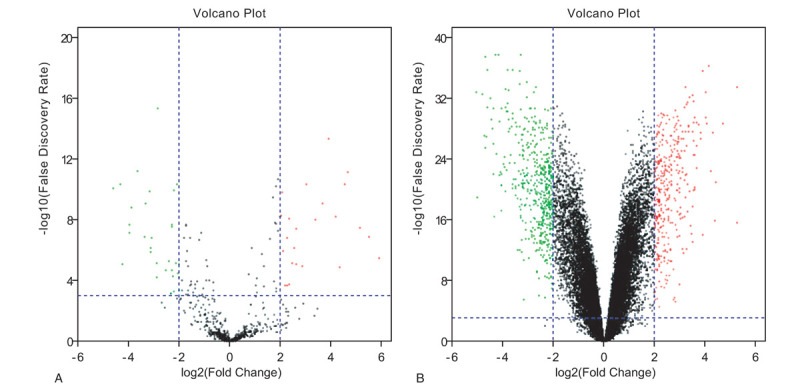
Volcano plot of the differentially expressed (DE) miRNAs (A) and DE-genes (B). (A) The black dots represent miRNAs that are not differentially expressed between 8 human normal lung samples and 35 human small cell lung cancer samples, and the red dots and green dots represent the upregulated and downregulated miRNAs in human small cell lung cancer samples, respectively. (B) The black dots represent genes that are not differentially expressed between 43 human normal lung samples and 15 human small cell lung cancer samples, and the red dots and green dots represent the upregulated and downregulated genes in human small cell lung cancer samples, respectively.

To identify DE-miRNAs and DE-genes from GSE19945 and GSE40275, respectively, we conducted normalized and differential expression analysis using limma software package, data before and after normalization were shown in Figure 1. Based on this analysis and our screening criteria, a total of 56 miRNAs were found to be significantly differentially expressed in human SCLC samples when compared to human normal lung samples, including 24 upregulated and 32 downregulated miRNAs. Furthermore, amount to 861 significantly differentially expressed genes were discovered, of which 350 upregulated and 511 downregulated. For better visualization, heatmap of DE-genes were provided in Figure 2, volcano plots of these DE-miRNAs and DE-genes were provided in Figure 3, the top 20 most upregulated and top 20 most downregulated miRNAs and genes were ranked by |fold change (FC)| in Tables 2–5.

**Table 2:**
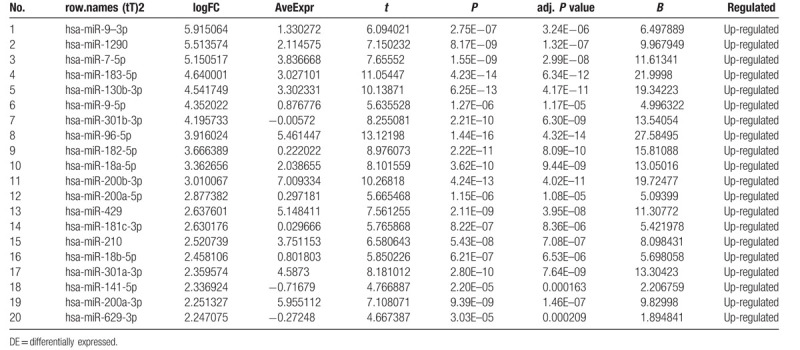
The top 20 upregulated DE-miRNAs (ranked by |fold change [FC]|).

**Table 3:**
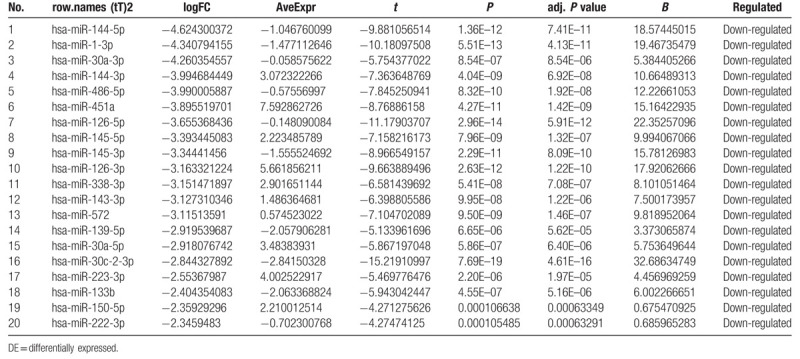
The top 20 downregulated DE-miRNAs (ranked by |fold change [FC]|).

**Table 4:**
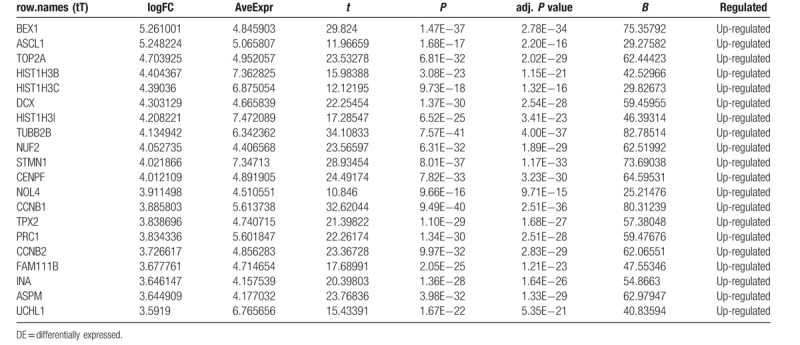
The top 20 upregulated DE-genes (ranked by |fold change [FC]|).

**Table 5:**
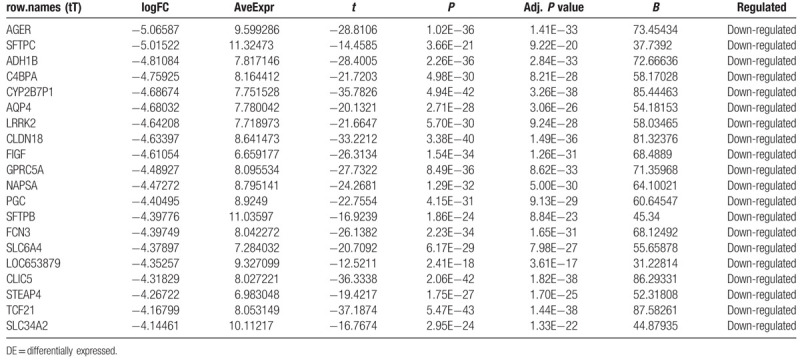
The top 20 downregulated DE-genes (ranked by |fold change [FC]|).

### 3.3 The target genes of DE-miRNAs

**Figure 4:**
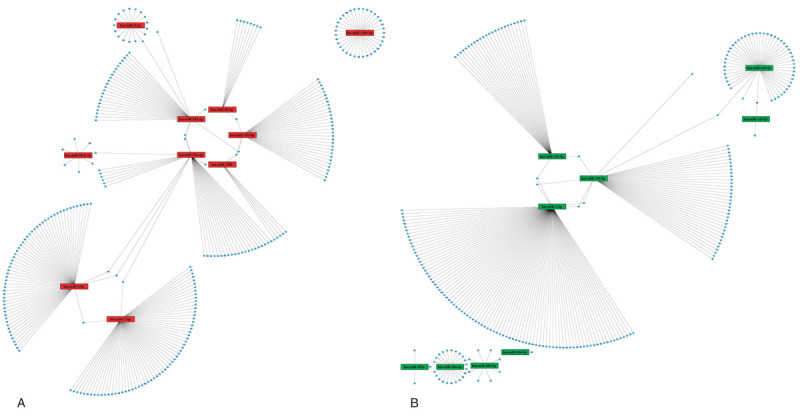
miRNAs-genes networks of top 10 upregulated and downregulated DE-miRNAs. (A) The network of the top 10 upregulated DE-miRNAs and their targets. (B) The network of the top 10 downregulated DE-miRNAs and their targets, because the 10th downregulated DE-miRNA hsa-miR-126-3p cannot find the same predicted target gene in the databases at the same time, so there is just nine genes in (B). DE = differentially expressed.

The top 10 upregulated DE-miRNAs were hsa-miR-9–3p, hsa-miR-1290, hsa-miR-7–5p, hsa-miR-183–5p, hsa-miR-130b-3p, hsa-miR-9–5p, hsa-miR-301b-3p, hsa-miR-96–5p, hsa-miR-182–5p, and hsa-miR-18a-5p. The top 10 downregulated DE-miRNAs were hsa-miR-144–5p, hsa-miR-1–3p, hsa-miR-30a-3p, hsa-miR-144–3p, hsa-miR-486–5p, hsa-miR-451a, hsa-miR-126–5p, hsa-miR-145–5p, hsa-miR-145–3p, hsa-miR-126–3p. We searched for the above miRNAs target genes in databases miRTarBase, TargetScan and miRDB respectively. The genes predicted by the three databases at the same time were identified as target genes for DE-miRNAs. Finally, 297 miRNAs-targets relationships (284 unique genes) were simultaneously predicted by three databases as the target of top 10 upregulating DE-miRNAs. In the same way, 245 miRNAs-targets relationships (238 unique genes) were identified as the target of top 10 downregulating DE-miRNAs. For better visualization, miRNAs-genes network of these DE-miRNAs were provided in Figure 4,

## 4 Enrichment analysis

### 4.1 Pathway and process enrichment analysis

**Figure 5:**
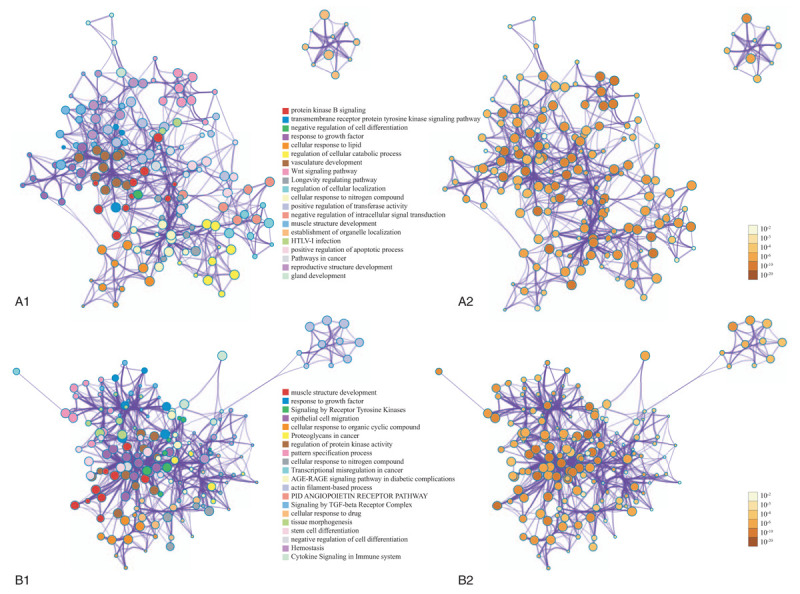
The visualized networks of enriched predicted target genes of top 10 upregulated and downregulated DE-miRNAs. (A) The visualized networks of enriched predicted target genes of top 10 upregulated DE-miRNAs, where each node represents an enriched term and is colored first by its cluster ID (A1) and then by its *P*-value (A2). (B) The visualized networks of enriched predicted target genes of top 10 downregulated DE-miRNAs, where each node represents an enriched term and is colored first by its cluster ID (B1) and then by its *P*-value (B2). DE = differentially expressed.

**Figure 6:**
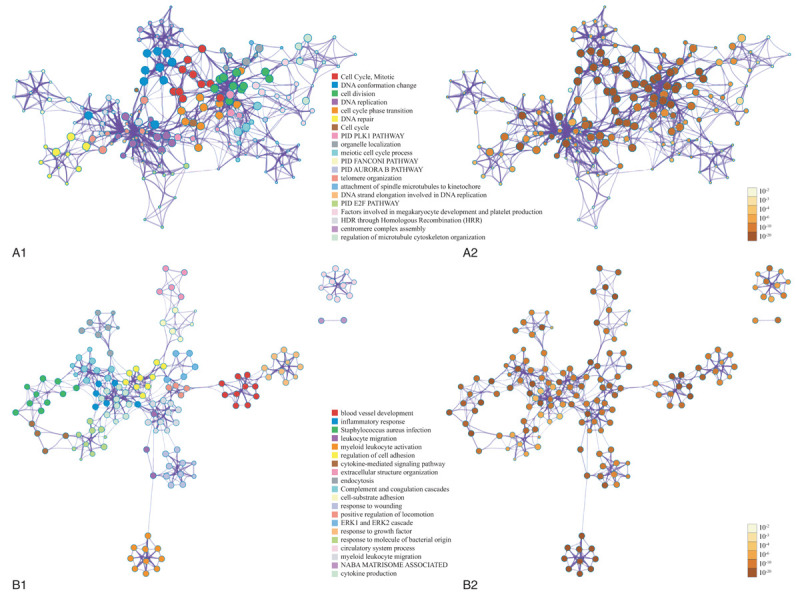
The visualized networks of enriched upregulated and downregulated DE-genes. (A) the visualized networks of upregulated DE-genes, where each node represents an enriched term and is colored first by its cluster ID (A1) and then by its *P*-value (A2). (B) The visualized networks of downregulated DE-genes, where each node represents an enriched term and is colored first by its cluster ID (B1) and then by its *P*-value (B2). DE = differentially expressed.

For the predicted target genes of DE-miRNAs and the DE-genes, pathway and process enrichment analysis have been carried out with the following ontology sources: Kyoto Encyclopedia of Genes and Genomes (KEGG) Pathway, Gene Ontology (GO) Biological Processes, Reactome Gene Sets, Canonical Pathways, and CORUM. The top 5 Pathway and Process Enrichment Analysis for target genes of the 10 upregulated DE-miRNAs included protein kinase B signaling, transmembrane receptor protein tyrosine kinase signaling pathway, negative regulation of cell differentiation, response to growth factor, cellular response to lipid. All of the above belong to the GO Biological Processes category. The top 5 Pathway and Process Enrichment Analysis for target genes of the top 10 downregulated DE-miRNAs included muscle structure development, response to growth factor, Signaling by Receptor Tyrosine Kinases, epithelial cell migration, cellular response to organic cyclic compound. Except for Signaling by Receptor Tyrosine Kinases (Reactome Gene Sets category), all above belong to the GO Biological Processes category. The top 5 Pathway and Process Enrichment Analysis for the upregulated DE-genes included Cell Cycle (Mitotic), DNA conformation change, cell division, DNA replication and cell cycle phase transition. They all belong to the GO Biological Processes category, except that Cell Cycle (Mitotic) belongs to Reactome Gene Sets category. The top 5 Pathway and Process Enrichment Analysis for the downregulated DE-genes included blood vessel development, inflammatory response, *Staphylococcus aureus* infection, leukocyte migration and myeloid leukocyte activation. Only *S aureus* infection belongs to the KEGG Pathway category, others belong to GO Biological Processes. For more details on Pathway and Process Enrichment Analysis, see Tables 6–9. To further capture the relationships between the terms, a subset of enriched terms have been selected and rendered as a network plot, where terms with a similarity >0.3 are connected by edges. We select the terms with the best *P*-values from each of the 20 clusters, with the constraint that there are no more than 15 terms per cluster and no more than 250 terms in total. The networks of enriched terms are visualized using Cytoscape (Figs. 5 and 6), where each node represents an enriched term and is colored first by its cluster ID (Fig. 5A1 and B1, Fig. 6A1 and B1) and then by its *P*-value (Fig. 5A2 and B2, Fig. 6A2 and B2).

**Table 6:**
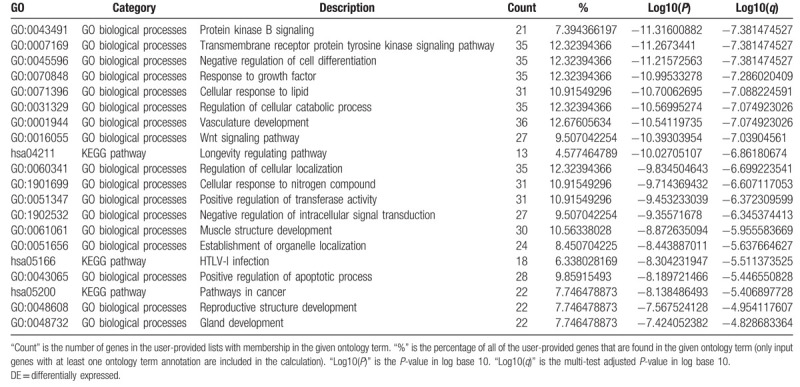
Top 20 clusters with their representative enriched terms of the targets genes of upregulated DE-miRNAs (one per cluster).

**Table 7:**
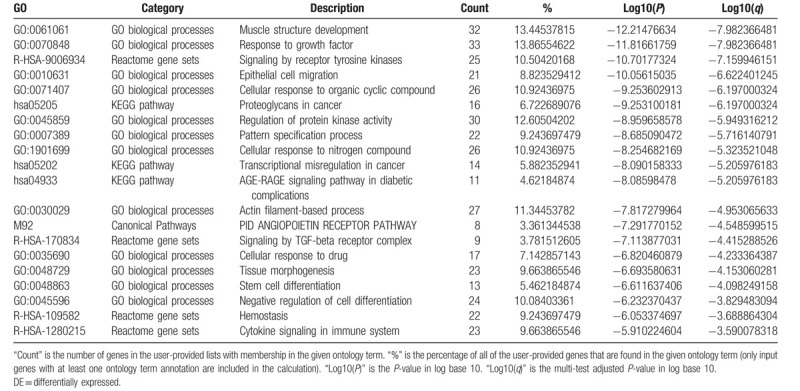
Top 20 clusters with their representative enriched terms of the targets genes of downregulated DE-miRNAs (one per cluster).

**Table 8:**
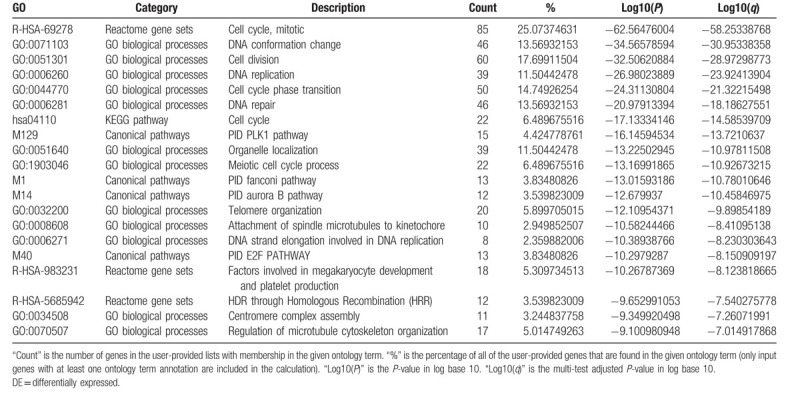
Top 20 clusters with their representative enriched terms of the upregulated DE-genes (one per cluster).

**Table 9:**
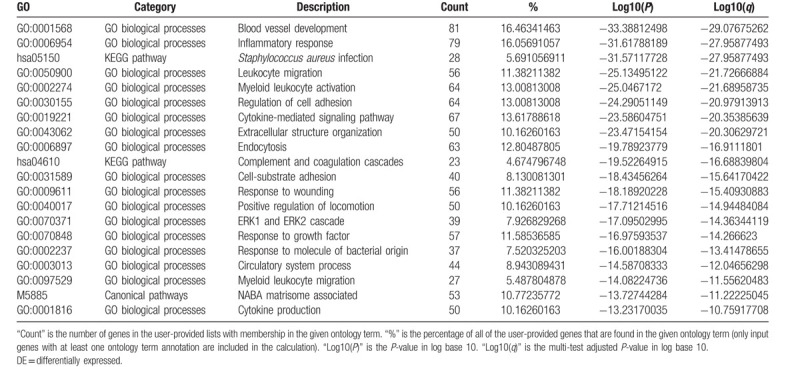
Top 20 clusters with their representative enriched terms of the downregulated DE-genes (one per cluster).

### 4.2 Protein–protein interaction enrichment analysis

**Figure 7:**
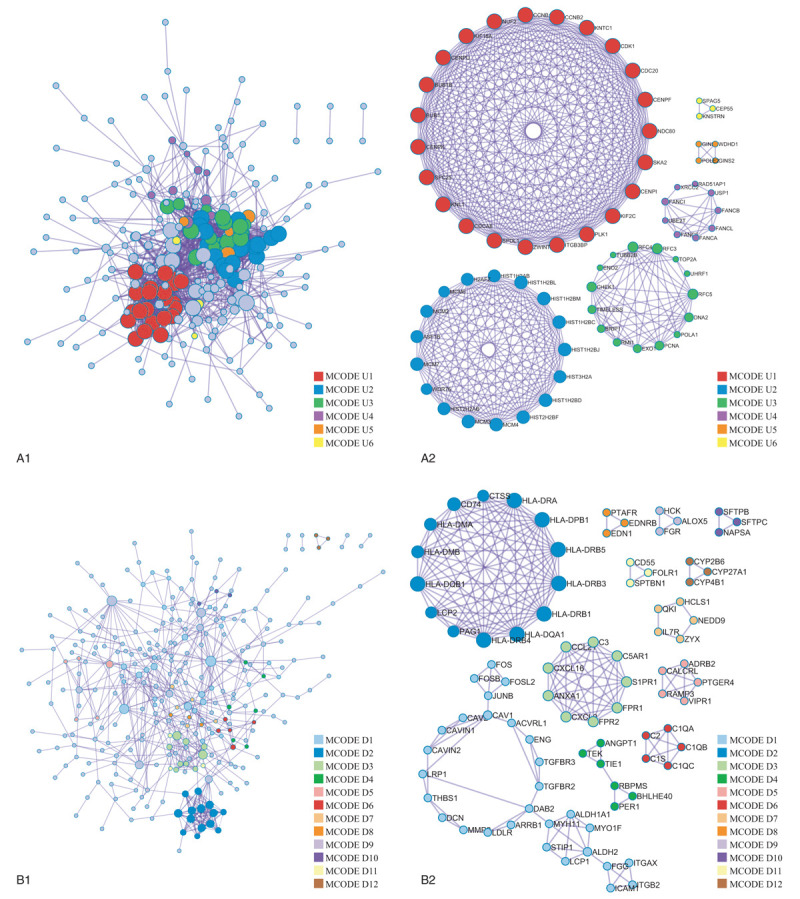
Visualized protein–protein interaction enrichment analysis of DE-genes. (A) Visualized protein–protein interaction enrichment of upregulated DE-genes. (B) Visualized protein–protein interaction enrichment of downregulated DE-genes. If the network contains between 3 and 500 proteins, the Molecular Complex Detection (MCODE) algorithm has been applied to identify densely connected network components. The MCODE networks identified for individual gene lists have been gathered and are shown in (A2) or (B2). DE = differentially expressed.

For the upregulated and downregulated DE-genes, protein–protein interaction enrichment analysis has been carried out with the following databases: BioGrid, InWeb_IM, OmniPath. The resultant network contains the subset of proteins that form physical interactions with at least one other member in the list. If the network contains between 3 and 500 proteins, the Molecular Complex Detection (MCODE) algorithm has been applied to identify densely connected network components. The MCODE networks identified for the DE-genes have been gathered and are shown in Figure 7.

Pathway and process enrichment analysis has been applied to each MCODE component independently, and the three best-scoring terms by *P*-value have been retained as the functional description of the corresponding components, shown in Tables 10 and 11 underneath corresponding network plots within Figure 7.

**Table 10:**
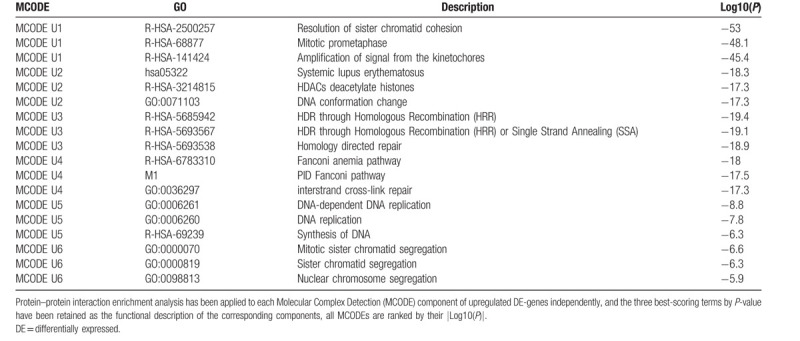
Protein–protein interaction enrichment analysis.

**Table 11:**
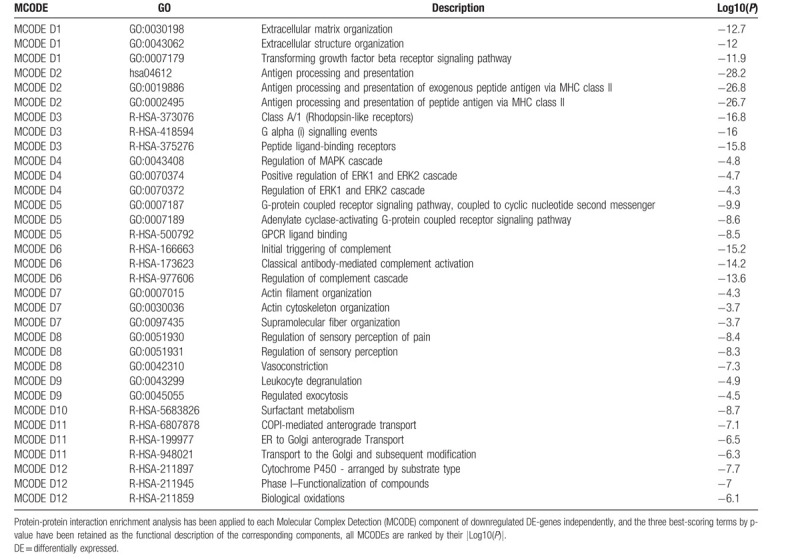
Protein-protein interaction enrichment analysis.

### 4.3 Conjoint analysis of DE-miRNAs and DE-genes

**Figure 8:**
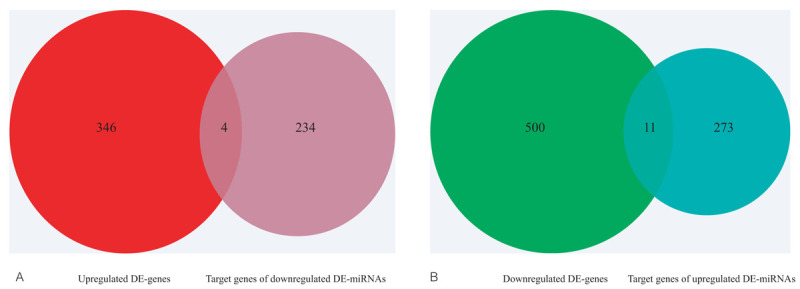
The intersections of DE-genes and the target genes of DE-miRNAs. (A) The intersection of upregulated DE-genes and the target genes of downregulated DE-miRNAs. (B) The intersection of downregulated DE-genes and the target genes of upregulated DE-miRNAs. DE = differentially expressed.

We got four upregulated and eleven downregulated DE-genes and their upstream miRNAs in this step. The downregulated DE-genes and their upstream miRNAs are as below, RBMS3 (RNA Binding Motif Single Stranded Interacting Protein 3)-hsa-miR-7–5p, NEDD9 (Neural Precursor Cell Expressed, Developmentally Down-Regulated 9)-hsa-miR-18a-5p, CRIM1 (Cysteine Rich Transmembrane BMP Regulator 1)-hsa-miR-18a-5p, TGFBR2 (Transforming Growth Factor Beta Receptor 2)-hsa-miR-9–5p, MYO1C (Myosin IC)-hsa-miR-9–5p, KLF4 (Kruppel Like Factor 4)-hsa-miR-7–5p, EMP2 (Epithelial Membrane Protein 2)-hsa-miR-1290, TMEM2 (Cell Migration Inducing Hyaluronidase 2)-hsa-miR-18a-5p, CTGF (Cellular Communication Network Factor 2)-hsa-miR-18a-5p, TNFAIP3 (TNF Alpha Induced Protein 3)-hsa-miR-18a-5p, THBS1 (Thrombospondin 1)-hsa-miR-182–5p. We found the upregulated DE-genes and their upstream miRNAs, KPNA2 (Karyopherin Subunit Alpha 2)-hsa-miR-144–3p, GPR137C (G Protein-Coupled Receptor 137C)- hsa-miR-1–3p, GRIK3 (Glutamate Ionotropic Receptor Kainate Type Subunit 3)-hsa-miR-144–3p and MTHFD2 (Methylenetetrahydrofolate Dehydrogenase (NADP+ Dependent) 2, Methenyltetrahydrofolate Cyclohydrolase)-hsa-miR-30a-3p. The conjoint analysis was more convinced (Fig. 8). Details are shown in Tables 12 and 13.

**Table 12:**
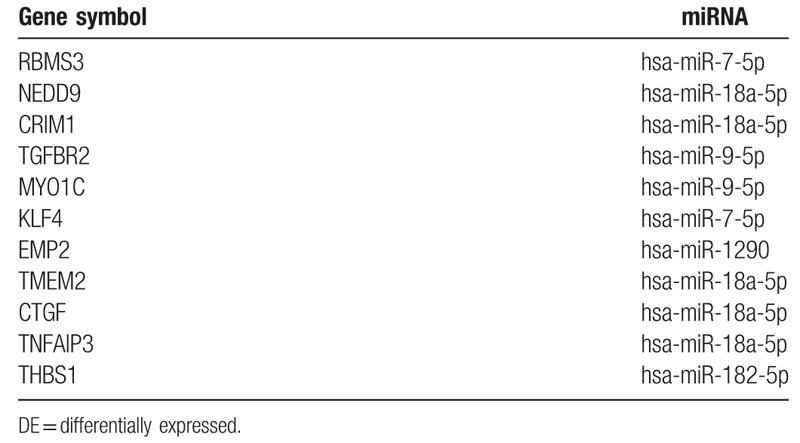
More convinced downregulated DE- genes and their upstream miRNAs.

**Table 13:**
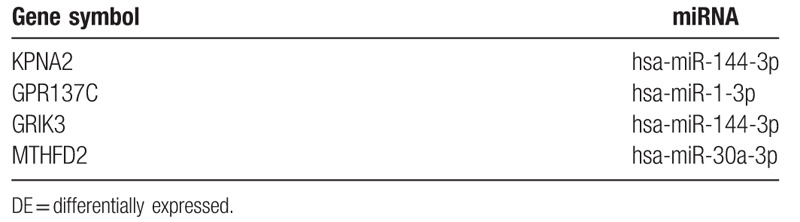
More convinced upregulated DE- genes and their upstream miRNAs.

## 5 Discussion

Small-cell lung carcinoma has long been divided into two clinicopathological stages, including limited stage and extensive stage. The stage is generally determined by the presence or absence of metastases, whether or not the tumor appears limited to the thorax, and whether or not the entire tumor burden within the chest can feasibly be encompassed within a single radiotherapy portal. In general, if the tumor is confined to one lung and the lymph nodes close to that lung, the cancer is said to be limited stage. If the cancer has spread beyond that, it is said to be extensive stage.^[14]^ In patients with extensive stage, median survival of 6 to 12 months is reported with currently available therapy, but long-term disease-free survival is rare.^[2]^ Regardless of stage, the current prognosis for patients with SCLC is unsatisfactory despite improvements in diagnosis and therapy made during the past 25 years. Without treatment, SCLC has the most aggressive clinical course of any type of pulmonary tumor, with median survival from diagnosis of only 2 to 4 months. About 10% of the total population of SCLC patients remains free of disease during the 2 years from the start of therapy, which is the time period during which most relapses occur. Even these patients, however, are at risk of dying from lung cancer (both small and non-small cell types).^[15]^ The overall survival at 5 years is 5% to 10%.^[15–18]^

In the medicine field, gene therapy is the therapeutic delivery of nucleic acid into a patient's cells as a drug to treat disease.^[19]^ The first attempt at modifying human DNA was performed in 1980 by Martin Cline, but the first successful nuclear gene transfer in humans, approved by the National Institutes of Health, was performed in May 1989.^[20]^ The possibility of correcting defective genes and modulating gene expression through gene therapy has emerged as a promising treatment strategy for cancer.^[21]^ Furthermore, the relevance of tumor immune microenvironment in supporting the oncogenic process has paved the way for novel immunomodulatory applications of gene therapy.^[21]^ Gene editing is a potential approach to alter the human genome to treat genetic diseases, viral diseases, and cancer.^[22–24]^ The first commercial gene therapy, Gendicine, was approved in China in 2003 for the treatment of certain cancers.^[25]^ Finding differential genes in cancerous and normal tissues is particularly important, but current genetic differences between SCLC and normal lung have rarely been reported.

miRNAs are defined as small non-coding RNAs with a length of about 20 to 25 nucleotides, which exert pivotal effects in various biological processes.^[26]^ miRNAs can regulate gene expression at post-transcriptional level through 3’-untranslated region (3’ UTR) pairing with target gene mRNAs.^[27]^ miRNAs regulate various targets which have critical roles in a wide spectrum of biological processes, including tumorigenesis and development, cell proliferation, metastasis, invasion, and apoptosis.^[28]^ therefore, miRNA can be used as a potential and effective target for the diagnosis and treatment of a variety of tumors. In addition, the role of miRNA in the diagnosis and treatment of cancer provides a new strategy in the field of cancer therapy.^[29,30]^ miR-30a-5p has been reported to inhibit the cell proliferation, invasion, migration, and autophagy in some types of tumors.^[31,32]^ Xiang Yang et al found that Inhibition of Beclin-1 by induction of miR-30a-5p may improve the therapeutic outcome via resensitizing the drug-resistant cells to chemotherapy in SCLC.^[33]^ Minting's team revealed that TSPAN12 promoted chemoresistance of SCLC under the regulation of miR-495.^[34]^ More and more abnormal expressed miRNAs in different tumors have been reported, but fewer reports in SCLC till now. Deeper research is still to be done to find more miRNAs with differential expression SCLC and normal lung.

In our study, we screened an eligible gene dataset and an eligible miRNA dataset in the GEO database, and obtained the DE-genes or DE-miRNAs for each dataset by bioinformatics analysis, in this step, we choose the FDR criteria as <0.001 in order to gain more credible difference and less noise in the following steps. Then we performed target gene prediction on the top 10 DE-miRNAs, the intersection of predicted target genes and DE-genes was taken as the final DE-genes. Then apply the prediction miRNAs-targets relationship of top 10 DE-miRNAs to the final DE-genes to gain more convincing DE-miRNAs, DE-genes and their one to one relationship.

From the results of the Pathway and Process Enrichment Analysis, it can be seen that protein kinase B signaling, muscle structure development, Cell Cycle (Mitotic) and blood vessel development are more likely to be involved in the development of SCLC. Protein kinase B signaling has been found to be involved in the survival and proliferation of a variety of tumor cells,^[35]^ including SCLC^[36,37]^ and non–small-cell lung cancer (NSCLC) cells.^[38]^ About muscle structure development, there are some of the more common paraneoplastic syndromes associated with lung cancer, such as syndrome of inappropriate anti-diuretic hormone (SIADH) and Nervous system problems, which will make muscle weakness or cramps.^[39]^ The cell cycle, the process by which cells progress and divide, lies at the heart of cancer.^[40]^ In cancer, as a result of genetic mutations, this regulatory process malfunctions, resulting in uncontrolled cell proliferation.^[40]^ Yi's study demonstrated that asparagine synthetase had an important role in the growth of human lung cancer cells by inhibiting the proliferation and arresting the cell cycle of lung cancer cells.^[41]^ Chengyu's team showed that Licochalcone A induces cell cycle arrest and apoptosis in lung cancer cells.^[42]^ blood vessel development, also known as angiogenesis, it is an essential component in the microenvironment to tumor growth and metastasis, whereas inhibiting angiogenesis has become a promising strategy for cancer therapy.^[43]^ VEGF overexpression and/or high VEGF serum levels have been reported both in non-small-cell lung carcinoma (NSCLC) and in SCLC.^[44]^

As we show them in Table 12, RBMS3, NEDD9, and CRIM1 are the top 3 downregulated genes in the small lung cancer tissue compared with normal lung. Their upstream miRNAs are hsa-miR-7–5p, hsa-miR-18a-5p, and hsa-miR-18a-5p, respectively. Recently, RBMS3 is found to be located at 3p24-p23, where is often found deleted or mutated in cancers, suggesting its potential role in tumor suppressing.^[45]^ Yanan's team found that RBMS3 is a tumor suppressor gene that acts as a favorable prognostic marker in lung squamous cell carcinoma.^[46]^ Chenglin reported that RBMS3 contributes to the tumorigenesis of lung adenocarcinoma.^[47]^ The upstream of RBMS3, hsa-miR-7-5p was reported to exert a tumor-suppressive function in glioblastoma and glioma by regulation of the EGFR, PI3K/ATK, Raf/MEK/ERK, and IGF-1R pathways.^[48]^ NEDD9 has been identified as a pro-metastasis gene in several types of cancers including melanoma and breast cancer.^[49]^ A recent article report that NEDD9 promotes lung cancer cell migration and invasion through the induction of epithelial-mesenchymal transition potentially via focal adhesion kinase activation.^[49]^ Shunsuke's study showed that NEDD9 plays a pivotal role in cell metastasis and invasion of NSCLC cells, and expression of NEDD9 appears to be a promising biomarker for NSCLC prognosis.^[50]^ The role of CRIM1 in controlling cancer cell behavior remains unknown.^[51]^ Losing Hui's group treated the non-SCLC line A549 with CRIM1 peptide or RNA interference, they found that CRIM1 could promote the migration and adhesion of cancer cells significantly.^[51]^ Turn to its upstream miRNA, hsa-miR-18a-5p can significantly reduce the hazard of dying for all cases, regardless of the tumor site.^[52]^

As in Table 13, KPNA2 (hsa-miR-144–3p), GPR137C (hsa-miR-1–3p), and GRIK3 (hsa-miR-144–3p) are the top 3 upregulated genes (upstream miRNAs) in the small lung cancer tissue compared with normal lung. KPNA2 was identified as a potential biomarker for non-small-cell lung cancer (NSCLC) by integration of the cancer cell secretome and tissue transcriptome.^[53]^ Xiaolei's study provided direct evidence to demonstrates that KPNA2 may contribute to nuclear translocation in lung cancer.^[54]^ So far, we have not found any research and reports on the GPR137C gene, which may become an innovation and hot spot for future research. The expression of GRIK3 was found in rhabdomyosarcoma, neuroblastoma, thyroid tumor, lung cancer, breast cancer, astrocytoma, multiple myeloma, glioma, and colorectal cancer.^[55]^ Meeta's team reported that GRIK3 gene was found to be methylated across all stages of lung adenocarcinoma, indicating that GRIK3 might be an epigenetic marker for diagnosis.^[56]^ hsa-miR-144–3p was demonstrated that markedly elevated in serum of patients with hepatocellular carcinoma.^[57]^ There is little literature on the relationship between hsa-miR-1–3p and cancer, and we believe this deserves more in-depth research.

Most of the six differential genes and four differential miRNAs we have derived from the study have been confirmed by previous studies to be associated with lung cancer or cancer. But there are still some differential genes and miRs have not been explored, which may be the innovation of future research. Our study may provide potentially likely regulators of SCLC invasion and metastasis can serve as biomarkers in SCLC, also can give future researchers a broader perspective and more inspiration. But it still has limitations:

1. target gene prediction was performed only on the top 10 DE-miRNAs;
2. Only target genes of the top 10 DE-miRNAs are selected for further enrichment analysis;
3. A lack of experimental verification, more studies should be performed.

## 6 Conclusion

In conclusion, we have successfully identified differential expression of genes (RBMS3, NEDD9, CRIM1, TGFBR2, MYO1C, KLF4, EMP2, TMEM2, CTGF, TNFAIP3, THBS1, KPNA2, GPR137C, GRIK3, and MTHFD2) and upstream miRNAs (hsa-miR-7–5p, hsa-miR-18a-5p, hsa-miR-18a-5p, hsa-miR-9–5p, hsa-miR-9–5p, hsa-miR-7–5p, hsa-miR-1290, hsa-miR-18a-5p, hsa-miR-18a-5p, hsa-miR-18a-5p, hsa-miR-182–5p, hsa-miR-144–3p, hsa-miR-1–3p, hsa-miR-144–3p, and hsa-miR-30a-3p) in SCLC based on bioinformatic analysis. At the same time, we found that the DE-genes (RBMS3, NEDD9, CRIM1, KPNA2, GPR137C, and GRIK3), hsa-miR-7–5p, hsa-miR-18a-5p, hsa-miR-144–3p, hsa-miR-1–3p, and the protein kinase B signaling, muscle structure development, Cell Cycle (Mitotic) and blood vessel development are highly likely to be related to the SCLC. Undoubtedly, continued efforts to delineate the mechanism of differential genes and miRNAs will reveal novel insights into SCLC.

## Acknowledgments

Thanks to the reviewers for their valuable comments and suggestions that helped improve the quality of our manuscript.

## Author contributions

**Resources:** Jian Zhang.

**Visualization:** Huan Luo.

**Writing – original draft:** Xiuwei Li, Chao Ma.

**Writing – review & editing:** Jinan Wang, Hongtao Guo.
